# Nightly sleep apnea severity in patients with atrial fibrillation: Potential applications of long-term sleep apnea monitoring

**DOI:** 10.1016/j.ijcha.2019.100424

**Published:** 2019-10-18

**Authors:** Dominik Linz, Mathias Baumert, Lien Desteghe, Kadhim Kadhim, Kevin Vernooy, Jonathan M. Kalman, Dobromir Dobrev, Michael Arzt, Manu Sastry, Harry J.G.M. Crijns, Ulrich Schotten, Martin R. Cowie, R. Doug McEvoy, Hein Heidbuchel, Jeroen Hendriks, Prashanthan Sanders, Dennis H. Lau

**Affiliations:** a Centre for Heart Rhythm Disorders (CHRD), South Australian Health and Medical Research Institute (SAHMRI), University of Adelaide and Royal Adelaide Hospital, Adelaide, Australia; b Department of Cardiology, Maastricht University Medical Centre, Maastricht, the Netherlands; c Department of Cardiology, Radboud University Medical Centre, Nijmegen, the Netherlands; d University Maastricht, Cardiovascular Research Institute Maastricht (CARIM), the Netherlands; e University of Adelaide, School of Electrical and Electronic Engineering, Adelaide, Australia; f Faculty of Medicine and Life Sciences, Hasselt University, Hasselt, Belgium; g Heart Centre Hasselt, Jessa Hospital, Hasselt, Belgium; h Department of Cardiology, Royal Melbourne Hospital and Department of Medicine, University of Melbourne, Melbourne, Australia; i Institute of Pharmacology, West German Heart and Vascular Centre, University Duisburg-Essen, Essen, Germany; j Department of Internal Medicine II, University Medical Centre Regensburg, Regensburg, Germany; k Academic Sleep Centre (CIRO+), Horn, the Netherlands; l National Heart and Lung Institute, Imperial College London (Royal Brompton Hospital), London, England, UK; m Adelaide Institute for Sleep Health (AISH), College of Medicine and Public Health, Flinders University and Sleep Health Service, Respiratory and Sleep Services, Southern Adelaide Local Health Network, Adelaide, Australia; n University of Antwerp and Antwerp University Hospital, Edegem, Belgium

## Abstract

In patients with atrial fibrillation (AF), the prevalence of moderate-to-severe sleep-disordered breathing (SDB) ranges between 21% and 72% and observational studies have demonstrated that SDB reduces the efficacy of rhythm control strategies, while treatment with continuous positive airway pressure lowers the rate of AF recurrence. Currently, the number of apneas and hypopneas per hour (apnea-hypopnea-index, AHI) determined during a single overnight sleep study is clinically used to assess the severity of SDB. However, recent studies suggest that SDB-severity in an individual patient is not stable over time but exhibits a considerable night-to-night variability which cannot be detected by only one overnight sleep assessment. Nightly SDB-severity assessment rather than the single-night diagnosis by one overnight sleep study may better reflect the exposure to SDB-related factors and yield a superior metric to determine SDB-severity in the management of AF.

In this review we discuss mechanisms of night-to-night SDB variability, arrhythmogenic consequences of night-to-night SDB variability, strategies for longitudinal assessment of nightly SDB-severity and clinical implications for screening and management of SDB in AF patients.

## 1 Introduction

Atrial fibrillation (AF) is the most common sustained arrhythmia and is associated with significant morbidity, increased risk of stroke, reduced quality of life, and increased mortality [1]. Concomitant risk factors such as hypertension, obesity, metabolic syndrome and ageing lead to structural remodeling processes in the atria which contribute to the progressive nature of AF and the reduced efficacy of standard antiarrhythmic pharmacological and catheter-based rhythm control strategies [2], [3], [4].

Another emerging risk factor for AF is sleep-disordered breathing (SDB). In patients with AF, the prevalence of categorically diagnosed moderate-to-severe SDB ranges between 21% and 72%, and this variation in reported prevalence is due to the nature of the cohorts studied as well as the differing scoring criteria and thresholds used for defining SDB [5], [6]. Observational studies have demonstrated that SDB reduces the efficacy of catheter- and pharmacological- based rhythm control strategies, while treatment with continuous positive airway pressure (CPAP) lowers the rate of AF recurrence after electrical cardioversion and improves catheter ablation outcomes [7], [8], [9], [10]. Based on these data, international AF guidelines recommend screening for signs and symptoms of sleep apnea and CPAP treatment when evaluating patients for rhythm control to reduce AF-recurrence and improve AF treatment results [11].

In sleep medicine, the number of apneas and hypopneas per hour (apnea-hypopnea-index, AHI) determined during a single overnight sleep study is clinically used to assess the severity of SDB [12]. Patients are categorized into having SDB when the AHI is ≥5/h. The severity of SDB is further graded, albeit quite arbitrarily, into mild (AHI 5–15), moderate (AHI 15–30) and severe (AHI ≥ 30) [13]. However, recent studies suggest that SDB-severity in an individual patient is not stable over time but exhibits a considerable night-to-night variability which cannot be detected by only one overnight sleep assessment [14], [15], [16], [17], [18], [19]. Nights with more respiratory events (higher AHI) may be related to a higher AF risk compared to nights with less respiratory events (lower AHI). Therefore, nightly SDB-severity assessment rather than the single-night diagnosis by one overnight sleep study may better reflect the exposure to SDB-related factors and yield a superior metric to determine SDB-severity in the management of AF.

In this review we discuss mechanisms of night-to-night SDB variability, arrhythmogenic consequences of night-to-night SDB variability, strategies for longitudinal assessment of nightly SDB-severity and clinical implications for screening and management of SDB in AF patients.

## 2 Mechanisms of night-to-night SDB variability in AF

Traditionally, assessment of SDB-severity is based on the result of one overnight sleep study. However, SDB patients with and without concomitant cardiovascular disease show considerable intra-individual night-to-night variability in AHI [14], [15], [16], [17], [18], [19]. Importantly, the intra-individual short-term night-to-night variability is higher in patients with mild SDB than in those with moderate to severe SDB.

### 2.1 General mechanisms

**Fig. 1:**
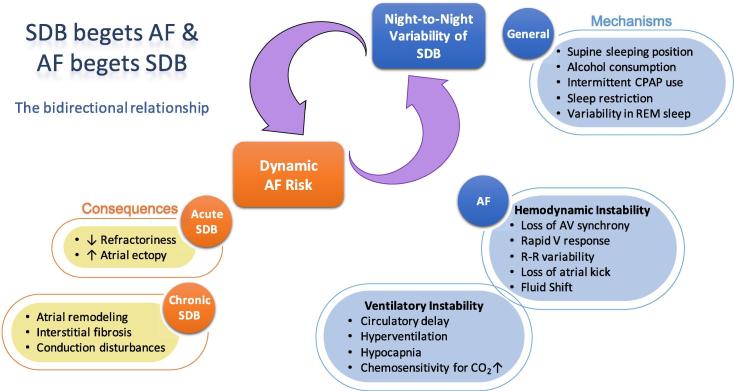
The hypothesized bidirectional relationship between sleep-disordered breathing (SDB) and atrial fibrillation (AF): SDB begets AF and AF begets SDB. Arrhythmogenic consequences of night-to-night variability in SDB severity (in orange) and general and AF-related mechanisms of night-to-night variability in SDB (in blue).

In the individual patient, between-night differences in SDB-severity may depend on positional factors such as time spent sleeping in the supine position. In positional obstructive sleep apnea, not just the position of the trunk, but also the degree of head rotation and flexion/extension can impact on the severity of SDB [20]; while elevation of the upper body can improve SDB-severity [21], [22]. SDB episodes predominantly occur during REM sleep in most patients. Sleep restriction with early termination of sleep (and thus less REM-sleep), sleep disturbances in general (first-night-effect in a sleep lab, jetlag) or intermittent non-use of antidepressants (with REM-rebound) may result in a variation in the amount of REM-sleep and may impact nightly SDB-severity. Further, intermittent CPAP use as well as alcohol consumption or use of hypnotics before bedtime may change arousability and contribute to night-to-night variability in SDB-severity (Fig. 1).

### 2.2 Mechanisms specific for patients with AF

During AF, loss of AV synchrony, a rapid ventricular response, a high beat-to-beat variability and the absence of atrial contraction (atrial kick) impair ventricular hemodynamics. Subsequently, the altered hemodynamics, which in turn can be variable on a nightly basis, can contribute to the dynamic SDB-severity. Emerging evidence points towards a crucial involvement of cardiovascular hemodynamics and nocturnal fluid shifts (due to variable fluid intake, use of diuretics and physical activity vs. sedentary behavior) in the genesis of particularly central but also obstructive respiratory events in AF patients (“AF begets SDB”) [23]. Non-anatomical factors such as arousability, loop gain and upper airway muscular responsiveness during sleep have been implicated in up to 56% of patients with obstructive sleep apnea and may contribute to night-to-night variability [24]. Increased sensitivity of peripheral and central chemoreceptors, pulmonary congestion and prolonged circulation time all contribute to the dysregulation of respiratory control and are associated with more prolonged events [6], [23], [25]. Accordingly, in patients with persistent AF, rhythm control by electrical cardioversion did not have an impact on the absolute AHI but reduced nocturnal central respiratory events and “unmasked” obstructive sleep apnea [26].

## 3 Arrhythmogenic consequences of night-to-night SDB variability in AF

Night-to-night variability in SDB-severity might result in a variable and dynamic exposure to SDB-related conditions which may impact the timing and extent of cardiovascular responses such as the onset of AF (“SDB begets AF”) (Fig. 1) [27]. Severe long-term SDB has been shown to be associated with structural remodeling in the atria in humans and animal models [28], [29], [30]. Mechanistically, obstructive respiratory events may cause structural remodeling and myocardial damage through repetitive mechanical atrial distension and atrial wall stretch as well as frequent episodes of oxyhemoglobin desaturation-resaturation. On top of the chronic structural alterations in the atria, transient apnea-associated autonomically-mediated shortening in action potential duration, increases in atrial ectopy and transient changes in conduction velocity create a dynamic arrhythmogenic substrate in the atria for enhanced AF susceptibility [31], [32], [33], [34], [35], [36]. This transient increase in atrial arrhythmogenesis may depend on nightly variability in SDB-severity and contribute to the temporal relationship between increased occurrence of arrhythmias during or after sleep apnea episodes [37], [38], [39], [40].

**Fig. 2:**
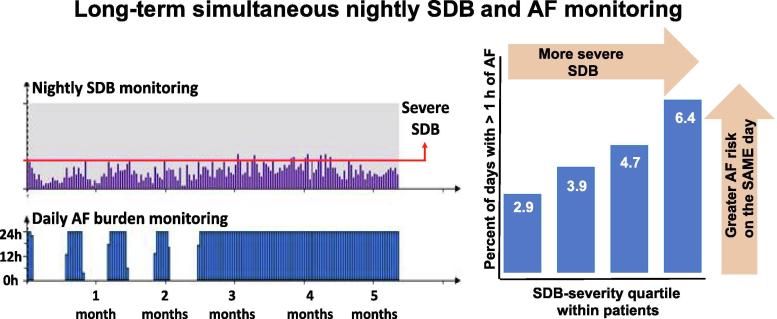
An example of long-term simultaneous nightly sleep-disordered breathing (SDB) and atrial fibrillation (AF) burden monitoring (left). Note the high night-to-night variability and AF episodes. Increasing daily AF risk dependent goes hand in hand with nightly SDB severity (right).

Longitudinally assessed SDB-severity (high SDB-burden), rather than the result of one overnight sleep study, may better reflect the exposure to SDB-related AF risk. In patients with implanted pacemakers, using simultaneous long-term night-by-night SDB and AF monitoring, we characterized night-to-night variability in SDB-severity and examined the relationship between SDB and AF with each patient acting as their own control (The VARIability in severity Of Sleep Apnea and nightly dynamic Atrial Fibrillation risk (VARI-OSA-AF) study) [40], [41]. In individual patients, the nights with the highest SDB-severity conferred a more than 2.3-fold increased risk of having at least one hour of AF the same day compared to nights with the lowest SDB-severity (Fig. 2) [40]. This suggests that increased cardiac stress induced by even a single night with more severe SDB can establish conditions predisposing to AF. Importantly, the relationship between nights with more severe SDB and increased AF-risk held for all individuals irrespective of the clinical diagnosis of SDB and mean AHI [40]. This suggests that the relative SDB-severity can trigger AF episodes and this is irrespective of underlying SDB-severity category.

## 4 Assessment of nightly SDB-severity and long-term SDB-pattern

Given the described dynamic relationship between nightly SDB-severity and AF risk, there exists a need for a metric that better captures the SDB ‘burden’ than a single overnight sleep study. Therefore, to assess nightly SDB-severity and long-term SDB-pattern (i.e. night-to-night variability in SDB), continuous long-term SDB monitoring (or monitoring of an appropriate surrogate parameter) is required. This is particularly relevant for patients in whom a high clinical suspicion of significant SDB exists but who, on a single ‘snapshot’ sleep study, do not exhibit the number of apneic or hypopneic events per hour required for a diagnosis of moderate-to-severe SDB, given the management implications associated with this category. A single sleep study or possibly even several repeated sleep studies may not be able to capture the proportion of nights spent with severe SDB and long-term SDB-pattern

**Fig. 3:**
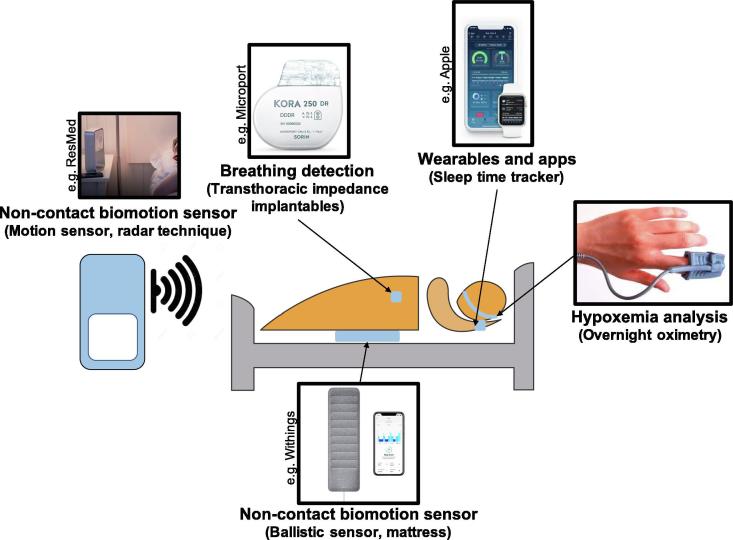
Different approaches to assess nightly sleep-disordered breathing (SDB) severity: Non-contact biomotion sensors (e.g. radar technique, ballistic sensors, etc.); breathing detection (e.g. transthoracic impedance changes recorded by implantable devices, etc); wearables and apps; hypoxia analysis (e.g. by overnight oximetry, etc).

In that regard, technologies implemented in implanted pacemaker devices that derive a surrogate marker of SDB, based on transthoracic impedance changes, are able to track fluctuations in tidal volume occurring during SDB events (Fig. 3) [42], [43], [44], [45]. The available algorithms have been validated against the AHI measured in polysomnography with variable sensitivity and specificity to detect severe SDB [42], [45]. Sleep apnea monitoring by pacemakers showed a significant night-to-night variability in SDB-severity [44] and pacemaker-detected SDB has been shown to predict new onset AF in a larger cohort [45]. Potentially, comparable technologies based on transthoracic impedance changes or accelerometer measurements could also be implemented in other implantable diagnostic devices such as loop recorders.

Non-contact biomotion sensors with actimetry, ballistic sensors or Doppler technology with radar frequencies can remotely monitor breathing during sleep (Fig. 3) [42], [43], [44]. A non‐contact biomotion radar sensor (SleepMinder™; ResMed) has been previously validated against AHI measured by polysomnography to monitor breathing during sleep remotely over periods of months to years [46], [47], [48]. Additionally, sleep tracking mats can monitor breathing disturbances during the night, and give users educational content about the signs of sleep apnea via a mobile app (Withings™; Health Mate app).

Wearable devices with inbuilt pulse oximeters are now also becoming commercially available raising the prospect of routinely measuring the night-to-night variability of SDB-severity by means of the oxygen desaturation index or determining hypoxic burden more broadly by newer validated algorithms (Fig. 3) [49], [50]. Additionally, computer analysis software of some long-term Holter systems can detect SDB by impedance changes of the surface ECG, although validation studies for long-term SDB assessment are not yet available.

Further, smartphone applications may be helpful for the purposes of large-scale, low-cost and long-term sleep monitoring (Fig. 3) [51]. Multiple applications have shown good capability in detection of sleep-wake cycles, but the reliability of smartwatch-based assessment of healthy and disturbed sleep and SDB-severity remains a key issue [52], [53]. Most of the available smartphone applications are not validated against the gold standard polysomnography and it remains unclear how monitoring could be organized in practice. However, apps can be helpful in applying dedicated in-app sleep coaching programs that can help to reduce fatigue, improve health, and support weight management efforts by leading towards a more balanced sleep schedule and managing sleep temperature, wake-up time etc.

## 5 Clinical implications

### 5.1 SDB screening

Given the high night-to-night variability, a single overnight sleep study may not be representative of true SDB-severity in a given individual. Particularly, in patients with mild-to-moderate SDB where the AHI is close to the somewhat ‘arbitrary’ cut-off values, misclassification of SDB-status may occur not infrequently. In patients with a high clinical suspicion of SDB, especially in the setting of treatment-resistant hypertension or AF recurrence despite initial success with rhythm control strategies, data from novel methods of SDB monitoring may prove to be helpful or repeat sleep studies should be considered.

### 5.2 SDB treatment adherence monitoring

Many patients use CPAP intermittently which results in an artificial night-to-night variability in SDB-severity. Long-term SDB monitoring could help to document therapeutic efficacy and compliance with CPAP and determine the biological response with respect to the reduction of nightly SDB-severity and AF-burden with treatment. Additionally, remote monitoring data of different CPAP machines could be made available to treating physicians (not just sleep specialists) to help assess and guide CPAP adherence. Although the appropriate AHI threshold at which to commence treatment remains unclear, a more effective reduction in nightly SDB-severity should result in a better reduction in SDB-related AF risk.

### 5.3 One-to-one comparisons of the SDB events and rhythm monitoring

Simultaneous SDB and AF monitoring may offer opportunities for a better understanding of how exactly SDB translates into higher AF risk. Potentially, the interaction between SDB variability and AF occurrence may help to distinguish patients where worsening in SDB-severity precedes AF episodes (“SDB begets AF”) from patients where AF episodes precedes changes in SDB-severity (“AF begets SDB“).

### 5.4 Clinical pathway and coordination of care

Assessment and management of SDB by continuous long-term combined AF and SDB-monitoring with a whole range of new technologies and disciplines may significantly alter how we practice SDB management in AF patients. The incorporation of these tools and data into the clinical work flow, as well as the empowering of patients through education regarding the rationale, tools (e.g. CPAP) and goals of their individualized SDB and AF treatment plans is crucial for treatment success [54]. A structured follow-up where assessment of AF burden, nightly SBD-severity, adherence and self-management of patients, as well as detection of side- and adverse effects of SDB treatment can be facilitated through a multidisciplinary approach with structured organization of care. Additionally, the utilization of monitoring tools allows informed patient-tailored management decisions. Long-term monitoring of SDB together with mobile health applications facilitates interactive feedback between patients and clinicians with respect to SDB disease progression, adherence to therapy or changes in SDB-severity upon interventions.

Additionally, SDB response to treatment or risk factor management interventions such as weight loss, exercise interventions, or behavioral interventions (sleep hygiene, alcohol abstinence, positional interventions etc.) could also be monitored by long-term sleep apnea recordings for more tailored goal directed management [55], [56], [57].

**Fig. 4:**
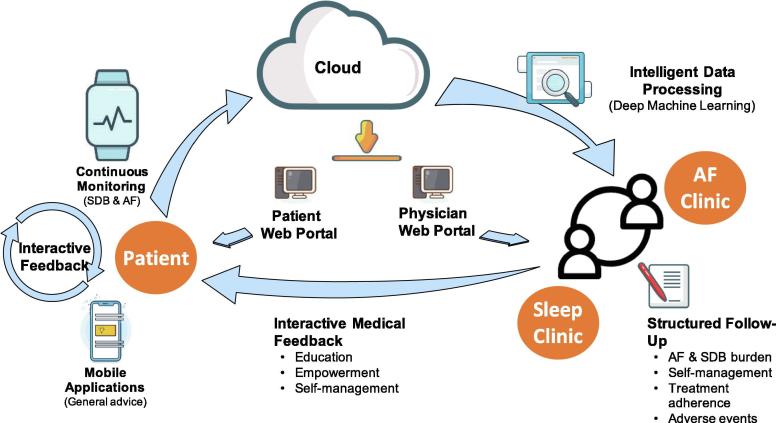
Long-term sleep-disordered breathing (SDB) monitoring: Basic principles of a possible clinical pathway to implement long-term SDB monitoring in an atrial fibrillation (AF) clinic. For more details, see text.

The basic principle of long-term SDB monitoring and a mobile SDB management system is shown in Fig. 4 and consists of the following modules: The patient end, which consists of a smartphone equipped with a special SDB app connected wirelessly to a long-term SDB monitoring device. The app provides general education and advice dependent on the measured SDB-severity. The acquired SDB data are transmitted and stored in a remote portal hosted in a health clinic or hospital. This portal is supported with intelligent data analytic tools (artificial intelligence, deep machine learning algorithms) that can process the data. These can be accessed and viewed by the patient and physician via two separate web portals, each designed and developed for separate access. Apps or home monitoring in the post treatment follow-up at home or as an adjunct to the sleep diary in the clinical setting may also improve treatment adherence compared with standard care by proactive management strategies [58]. Remote telemonitoring of SDB patients in terms of CPAP adherence, following SDB disease progression, AF burden and daily physical activity may be an important component of a comprehensive health monitoring and lifestyle coaching program [59].

## 6 Conclusions

Nightly SDB-severity, determined by long-term SDB monitoring, rather than the single-night diagnosis by one overnight sleep study, may better reflect SDB-associated AF risk. The ability to perform long-term monitoring may change how we manage SDB in AF patients. Future observational “real world” studies and prospective and randomized intervention studies are warranted to: (I) determine the best technology, feasibility, accuracy and cost-effectiveness of long-term SDB monitoring in the management SDB (II) affirm that treatment of “intermittent/paroxysmal SDB” leads to improved AF outcomes and (III) determine how long-term monitoring of SDB can be implemented as a component of structured risk factor management programs.

## Conflict of interest

The authors declare that there is a no conflict of interest.
